# Impact of COVID-19 Outbreak on the Gynecological Outpatients HPV Infection Rate in Wuhan, China: A Retrospective Observational Study

**DOI:** 10.3389/fmed.2022.799736

**Published:** 2022-04-11

**Authors:** Hang Liu, Qian Yao, Di Li, Zhiming Zhao, Yan Li

**Affiliations:** ^1^Department of Clinical Laboratory, Renmin Hospital of Wuhan University, Wuhan, China; ^2^Department of Geratology, Renmin Hospital of Wuhan University, Wuhan, China

**Keywords:** HPV, COVID-19, infection rate, impact, outbreak

## Abstract

**Background:**

The recent severe acute respiratory syndrome coronavirus 2 (SARS-CoV-2) outbreak has caused millions of deaths and greatly influenced the timely diagnosis and treatment of other diseases. Throughout the pandemic, there was a dramatic reduction in the prevalence of several sexually transmitted infections. However, the impact of the ongoing pandemic on human papillomavirus (HPV) infection rates has not been investigated thus far.

**Materials and Methods:**

We retrospectively collected data regarding HPV and cervical cancer screening results of outpatients from gynecological clinics of a tertiary hospital from 1 December 2018 to 31 December 2020 in Wuhan. Based on the timeline of the SARS-CoV-2 pandemic in Wuhan, we divided this period into four relatively independent stages to compare the HPV screening visit numbers and infection rates.

**Results:**

There was a 50% drop in HPV screening visits and a 10% drop in HPV infection rates throughout the pandemic when compared with the numbers collected before the pandemic. Strict lockdown measures greatly decreased the HPV infection rate (17.03 vs. 8.29, *P* = 0.003). During the pandemic, the most prevalent HPV genotypes were HPV 16, 52, 58, and 53. After the pandemic, the HPV infection rate recovered quickly, but it was still slightly lower than the infection rate found before the outbreak (23.3 vs. 21.2%).

**Conclusion:**

During coronavirus disease 2019 (COVID-19) pandemic, cervical cancer screening visits and HPV infection rates have decreased dramatically. The HPV transmission has also decreased after strict lockdown. Effective HPV and cervical cancer screening programs should be strengthened immediately to reduce the transmission of HPV during and after the pandemic.

## Introduction

Coronavirus disease 2019 (COVID-19) pandemic caused by the 2019 novel coronavirus [severe acute respiratory syndrome coronavirus 2 (SARS-CoV-2)] has caused millions of deaths and is still spreading in many countries around the world (https://covid19.who.int/). Strict quarantine and antimigration measures, keeping social distance, and vaccine injections have proved to be efficient approaches to stop the propagation of this pandemic. However, these strict lockdown measures and the restriction of bodily contact have led to negative influences on the psychological health and routine cancer screening and treatment (1–3). Nevertheless, these measures are also probably beneficial for the control of other seasonal respiratory virus infections and sexually transmitted infections (STIs). Indeed, throughout the SARS-CoV-2 pandemic, there was a dramatic decline in seasonal respiratory virus infections (4), while the incidence of several STIs, such as syphilis, chlamydia, and gonorrhea, has also decreased (5, 6).

Human papillomavirus (HPV) infection is a common STI that is recognized as the leading cause of cervical cancer. The WHO aims to eradicate cervical cancer by 2030 (7); however, this goal has been greatly affected by the SARS-CoV-2 pandemic (8, 9). HPV and cervical screening and HPV vaccine injections have been suspended by the quarantine and lockdown policies in many countries and regions. Considering the SARS-CoV-2 pandemic may positively affect the control of HIV and other STIs, we speculate that there is a similar influence on HPV infection. Wuhan is the first Chinese city that has undergone a large outbreak of SARS-Cov-2 and has since then thoroughly recovered from this pandemic, posing Wuhan as a favorable model to investigate the impact of the pandemic on HPV infection and screening.

Therefore, the purpose of this study was to investigate the impact of SARS-CoV-2 on HPV infection rates during and after the pandemic. These results can provide a basis to formulate improved HPV and cervical screening policies during and after the outbreak of SARS-Cov-2.

## Materials and Methods

### Study Design

**Figure 1:**
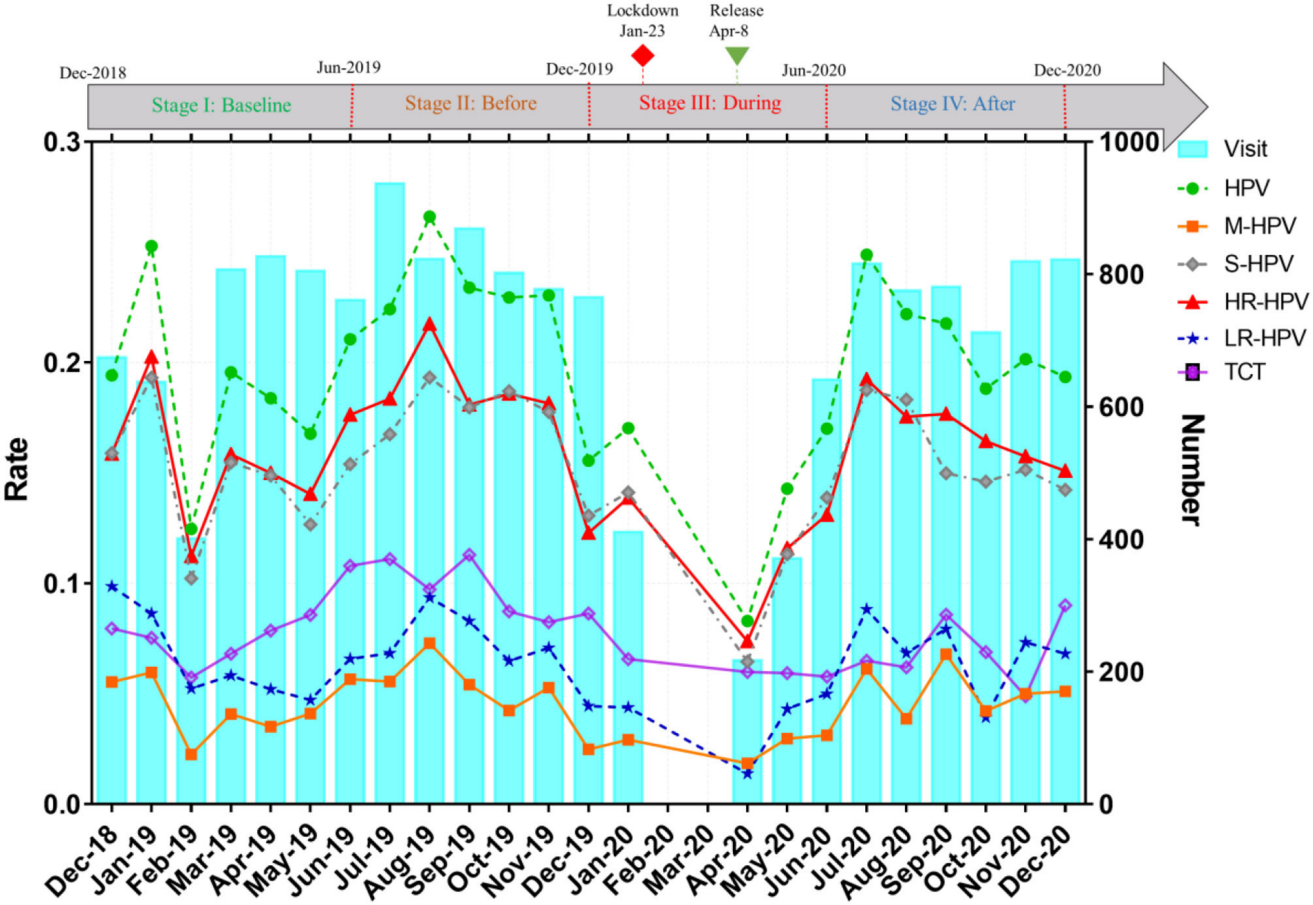
Overall trend of monthly HPV screening visits and infection rates. HPV, human papillomavirus; HR-HPV, high-risk HPV infection; LR-HPV, Low-risk HPV infection; M-HPV, multiple genotype HPV infection; S-HPV, single genotype HPV infection; TCT, ThinPrep cytology test abnormal. (i) Stage I (December 1, 2018–May 31, 2019): Baseline in the same period last year before the SARS-CoV-2 outbreak; (ii) Stage II (June 1, 2019–November 31, 2019): Before SARS-CoV-2 outbreak; (iii) Stage III (December 1, 2019–June 30, 2020): During the prevalence of SARS-CoV-2; (iv) Stage IV (July 1, 2020–December 31, 2020): After the SARS-CoV-2 pandemic.

We retrospectively collected data regarding HPV and cervical cancer screening results of outpatients from the gynecological clinic of the Renmin Hospital of Wuhan University from 1 December 2018 to 31 December 2020. The outpatients visited the clinic for many different reasons, including physical examination, vaginitis, cervicitis, undiagnosed abdominal pain, and irregular vaginal bleeding. Exclusion criteria included: <16 years of age, pregnancy, and a previous diagnosis of precancerous lesions or cervical cancer. According to the timeline of the SARS-CoV-2 outbreak in Wuhan, we divided our data collection period into four relatively independent stages (Figure 1). (i) Stage I (1 December 2018–31 May 2019): baseline of the same period 1 year before the SARS-CoV-2 outbreak; (ii) Stage II (1 June 2019–31 November 2019): right before the SARS-CoV-2 outbreak; (iii) Stage III (1 December 2019–30 June 2020): peak prevalence of SARS-CoV-2; and (iv) Stage IV (1 July 2020–31 December 2020): the period after the SARS-CoV-2 pandemic was contained.

### Human Papillomavirus Genotype and Cervical Cytology

Human papillomavirus genotyping and ThinPrep cytology (TCT) samples were collected as previously described (10). The HPV genotype was detected in the clinical laboratory, while the cervical cytology was processed within the pathology department. HPV genotype detection was performed according to the manufacturer's handbook (Tellgenplex^®^ HPV27 genotyping Assay). High-risk HPV (HR-HPV) infection was defined as an infection with HPV genotypes 16, 18, 26, 31, 33, 35, 39, 45, 51, 52, 53, 56, 58, 59, 66, 68, and/or 82. Low-risk HPV (LR-HPV) infection was defined as infection with HPV genotypes 6, 11, 40, 42, 43, 44, 55, 61, 81, and/or 83. Single HPV (S-HPV) infection was defined as an infection with only one HPV genotype. Multiple HPV (M-HPV) infection was defined as a co-infection with two or more HPV genotypes. Incident HPV infection was defined as the first detection of an HPV genotype in our hospital with a normal TCT result. Persistent HPV infection was defined as more than two consecutive positive HPV genotype test results in our hospital, or the first detection of an HPV genotype with an abnormal TCT result (11).

ThinPrep cytology samples were prepared by AZR-D and ATP-E liquid-based cytology machine (Xiaogan Aohua Medical Technology Corporation, Ltd,) following the manufacturer's instructions. Experienced cytopathologists made a pathological diagnosis for each TCT sample according to the 2002 Bethesda System for Reporting Cervical Cytology standard. Because of the relatively small numbers of atypical gland cell (AGC), squamous-cell carcinoma (SCC), and adenocarcinoma diagnostic results, and to better compare the TCT positive results among different stages, we classified and merged TCT abnormal results into three groups: 1. atypical squamous and cells of undetermined significance (ASCUS), high-grade ASCUS which included atypical squamous cells that we could not exclude a high-grade squamous intraepithelial lesion (ASCUS-H), and AGC, all of which were classified as the ASCUS group; 2. low-grade squamous intraepithelial lesion (LSIL), and 3. high-grade squamous intraepithelial lesion (HSIL), SCC, and adenocarcinoma, herein referred to as the HSIL group.

### Statistical Analysis

All the statistical analyses were conducted using SPSS (version 22.0, Chicago, Illinois, USA) and graphs were drawn using GraphPad Prism (version 8.0, San Diego, California, USA) or R software (version 3.6.1). Both the chi-squared test and the Fisher's exact test were used to compare categorical variables, which were expressed as frequency and percentage. According to whether the data were normally distributed, an ANOVA or the Kruskal–Wallis test was applied to compare continuous variables. A two-way *P* < 0.05 was considered as statistically significant. The Bonferroni correction was performed for multiple comparisons.

## Results

### Characteristics of the Study Population and Infection Rates

**Figure 2:**
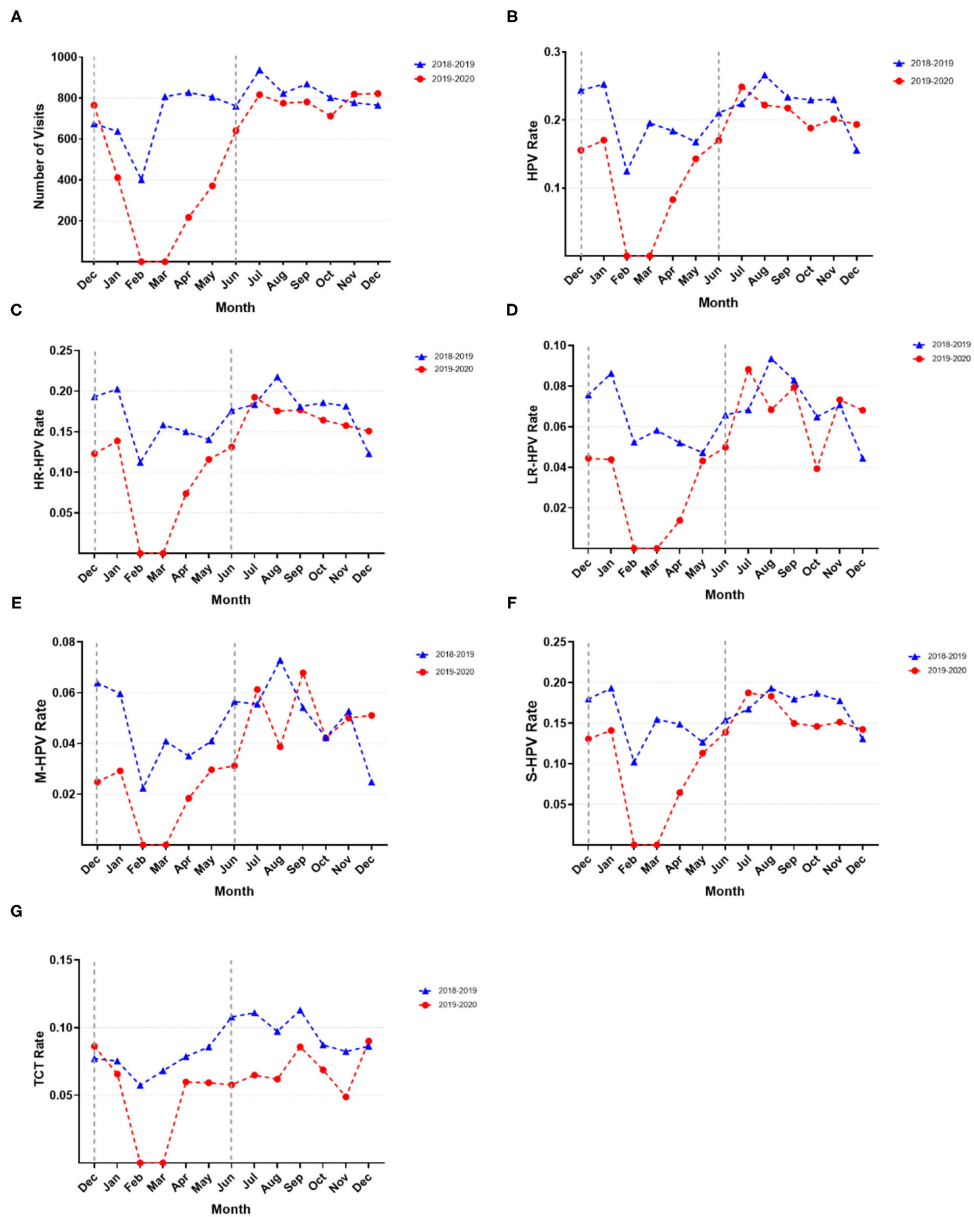
Monthly comparison of HPV screening visits and infection rates. **(A)** Visit number per month; **(B)** HPV infection rate; **(C)** HR-HPV infection rate; **(D)** LR-HPV infection rate; **(E)** M-HPV infection rate; **(F)** S-HPV infection rate; **(G)** TCT abnormal rate.

As shown in Figure 1, the gynecological HPV screening visits, the HPV, HR-HPV, LR-HPV, M-HPV, and S-HPV infection rates, and the abnormal TCT rate fluctuated slightly every month; however, these rates presented a rapid decline and ascent of a V-shaped curve from December 2019 to June 2020. Upon the beginning of the pandemic, the number of HPV screening visits decreased and stopped during the lockdown period. Upon city reopening, the number of HPV screening visits increased sharply. After the pandemic, the number of HPV visits nearly recovered to levels similar to those found before the outbreak; however, the HPV and HR-HPV infection rates presented a declining tendency. Interestingly, the HPV, HR-HPV, and S-HPV infection rates generally showed a similar upward or downward trend. Compared with the same period in 2018–2019, the gynecological HPV screening visits, HPV, HR-HPV, LR-HPV, M-HPV, and S-HPV infection rates showed a significant decline during COVID-19 outbreak in 2019–2020 (Figures 2A–G). After the pandemic (after July 2020), the number of HPV screening patients, HPV, HR-HPV, LR-HPV, M-HPV, and S-HPV infection rates nearly returned to the pre-pandemic level and remained relatively stable. Surprisingly, at the end of the study period (December 2020), we found that the number of visits, HPV, HR-HPV, LR-HPV, M-HPV, and S-HPV infection rates exceeded those of before the pandemic.

### Human Papillomavirus Infection Rates at Different Pandemic Stages

**Figure 3:**
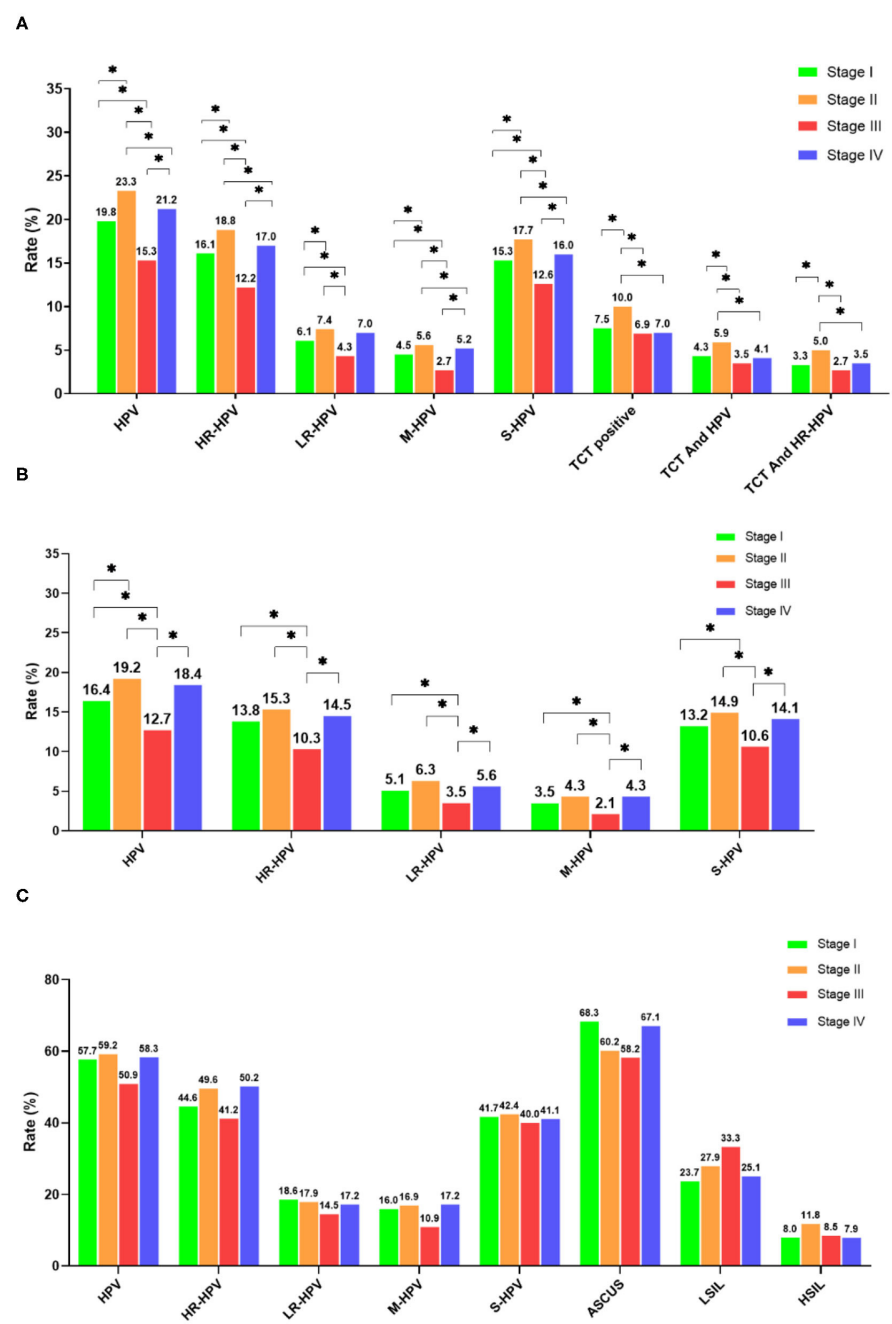
Comparison of HPV infection rates in different stages. **(A)** Overall population; **(B)** TCT normal population; **(C)** TCT abnormal population. *P < 0.05 and **P < 0.01.

The total number of HPV screening visits in stage III of the pandemic decreased by ~50% when compared to visits during stages II and IV. Meanwhile, the gynecological visits during stage IV returned quickly back to levels found during Stage II (Supplementary Table 1). Both the average age and stratified age groups of visits showed a similar distribution among these different stages. Differences with regard to HPV infection rates between the stages are shown in Figure 3A. The HPV, HR-HPV, and S-HPV rates significantly decreased in stage III when compared with those of stages I, II, and IV. Likewise, both the LR-HPV and M-HPV rates also declined dramatically in stage III when compared with those of other stages. Stage II presented remarkably higher infection rates than stages I and IV. Also, the TCT, concurrent TCT and HPV, and co-positive TCT and HR-HPV were the highest during stage II, despite a lack of statistical significance when compared with other stages.

**Figure 4:**
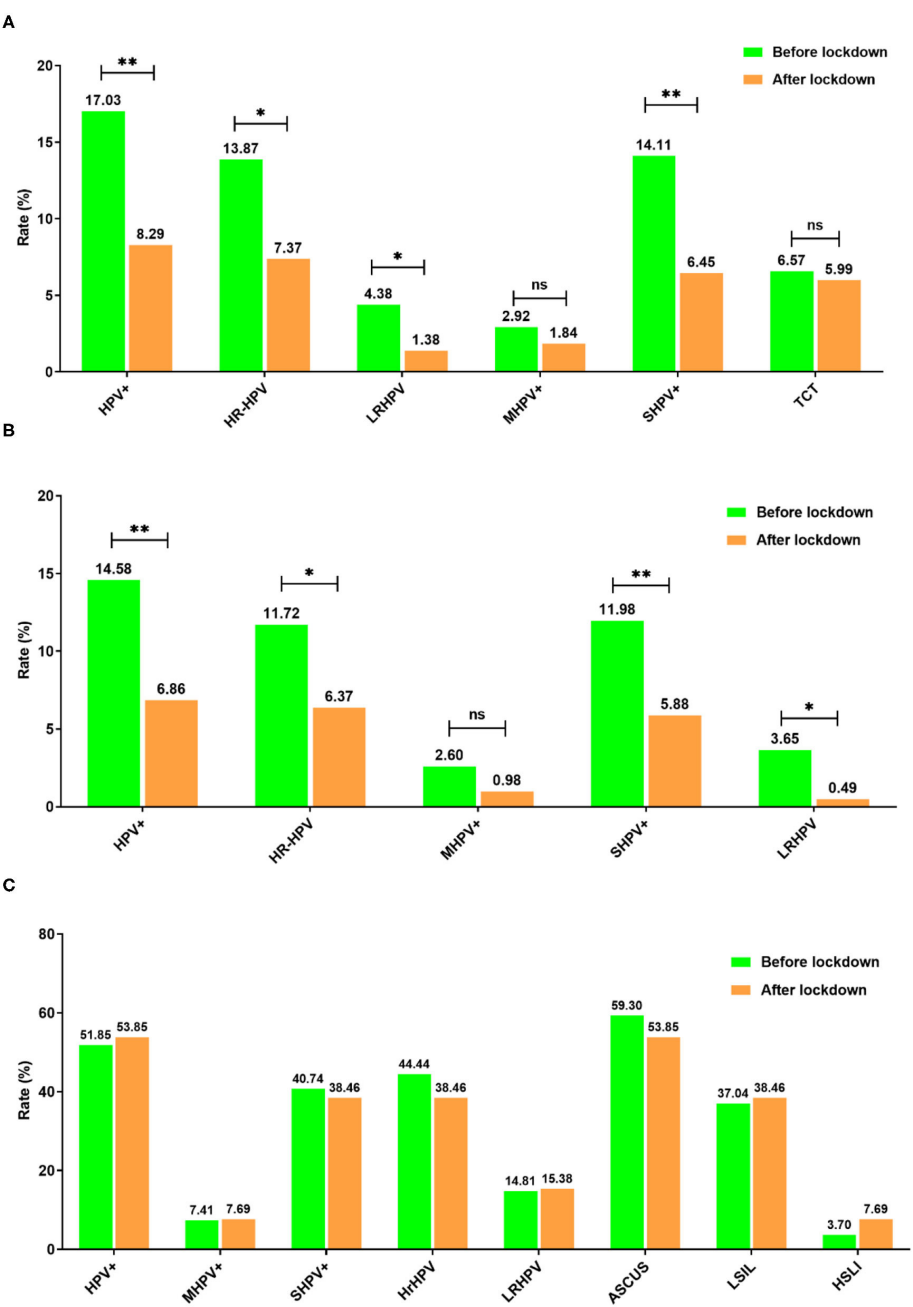
Comparison of month HPV infection rates before and after the lockdown period. **(A)** Overall population; **(B)** TCT normal population; **(C)** TCT abnormal population. *P < 0.05 and **P < 0.01.

Human papillomavirus infection in individuals with a normal TCT was considered as a mixture of the incident and persistent infections, while those with an abnormal TCT with a positive HPV infection were treated as a persistent infection. The average age between the four stages was similar, while HPV, HR-HPV, S-HPV, LR-MHV, and M-HPV rates presented downtrends in stage III for individuals with a normal TCT when compared with the other three stages (Supplementary Table 2, Figure 3B). However, for individuals with an abnormal TCT, the HR-HPV, S-HPV, LR-HPV, and M-HPV infection rates were similar across different stages (Supplementary Table 3, Figure 3C). In addition, HPV, HR-HPV, M-HPV, and S-HPV infection rates were significantly decreased after lockdown, while abnormal TCT rates did not significantly change (Figure 4A). Interestingly, the infection rates of HPV, HR-HPV, S-HPV, LR-HPV decreased in the normal TCT group, but were unchanged in individuals with an abnormal TCT (Figures 4B,C).

### Prevalence and Composition of Human Papillomavirus Genotypes

**Figure 5:**
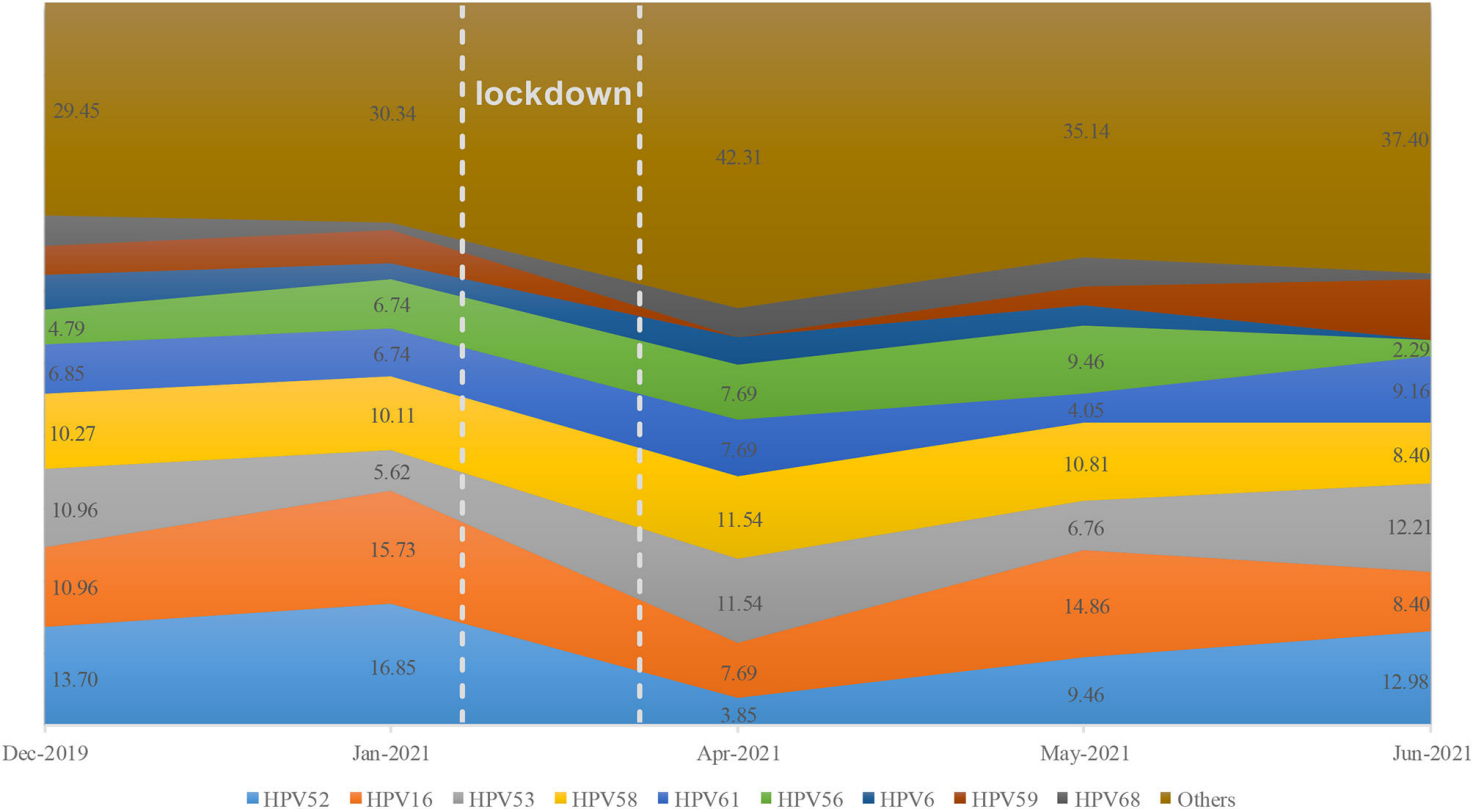
Monthly distribution of HPV genotypes during the SARS-CoV-2 pandemic.

**Figure 6:**
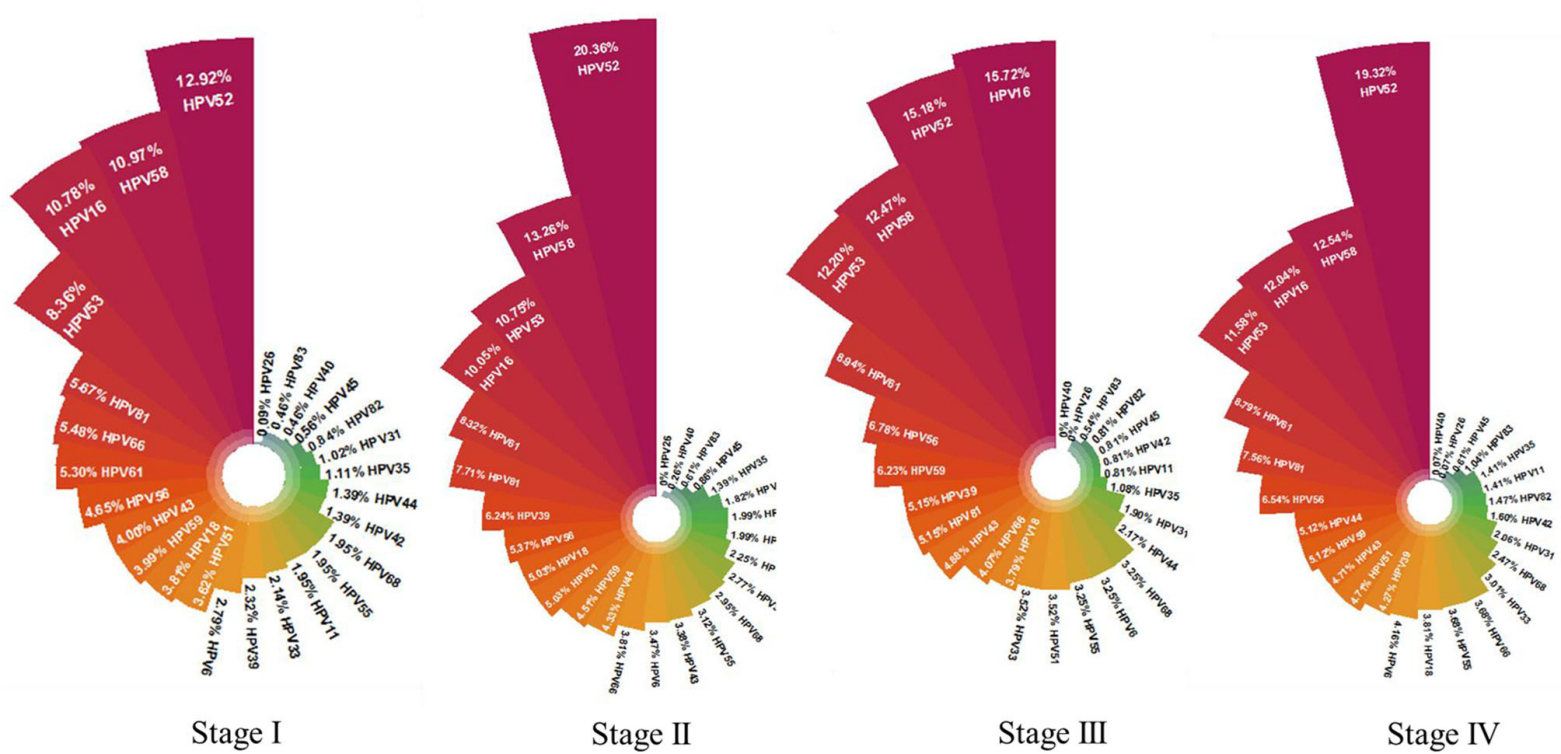
Prevalence and composition of HPV genotype in different stages. Stage I: Baseline in the same period last year before the SARS-CoV-2 outbreak; Stage II: Before SARS-CoV-2 outbreak; Stage III: During the prevalence of SARS-CoV-2; Stage IV: After the SARS-CoV-2 pandemic.

The monthly distribution of HPV genotypes greatly varied before and after the lockdown (Figure 5), which suggested that the composition of HPV genotypes was unstable during the pandemic. To further explore whether the distribution of HPV genotype changed during the pandemic, Stage III (seven months including before, during, and after the lockdown phase) was selected to calculate the composition of HPV genotypes during the pandemic. In the first, second, and fourth stages, the descending order of HPV genotype prevalence was HPV 52, 58, 16, and 53. This changed to HPV genotypes 16, 52, 58, and 53 during the pandemic (Figure 6). Compared before and after the pandemic, the distribution of HPV genotypes 52 and 58 changed in the overall population but did not vary in TCT positive individuals during the pandemic (Supplementary Table 4).

## Discussion

Human papillomavirus infection is the leading risk factor for the development of cervical cancer; therefore, HPV and cervical cancer routine screening are widely accepted to be the most effective measures for the early diagnosis and prevention of cervical cancer progression. With the SARS-CoV-2 outbreak and strict pandemic prevention measures, these procedures HPV and cervical cancer screening have been delayed or halted in many countries and regions. Several reports have described that SARS-CoV-2 has had a great influence on STIs infection rates (5, 12); however, there is still a lack of investigation on the impact of this pandemic on the HPV infection rate. This is the first study that has focused on whether SARS-CoV-2 impacted the prevalence of HPV infection.

In this study, during the SARS-CoV-2 outbreak, we observed a rapid decline and ascent (in a V-shaped curve) of the number of clinical visits. We speculate that the severity of the pandemic, lockdown measures, and insufficient medical resources are among the main reasons for the visit numbers to decrease this quickly. After the end of strict quarantine measures, clinical visits dramatically increased every month. The control and elimination of the pandemic would allow more patients to safely visit the hospital for clinical diagnosis and treatments. A report of the Kaiser Permanente Southern California (KPSC) program described HPV and cervical cancer screening rates decreased 80% during the SARS-CoV-2 pandemic. After the reopening of the cities, the cervical screening rates returned gradually to baseline (13).

In this study, HPV infection rates have dramatically decreased during the pandemic, consistent with previous studies focusing on different STIs (6, 14). These results suggest that the pandemic exerted a negative influence on HPV transmission and lockdown measures greatly decreased HPV dissemination. In addition, HPV infections among individuals with a normal TCT declined significantly during the pandemic, while there were no changes for patients with an abnormal TCT. Also, the HPV infection rate decreased after a lockdown in the TCT-negative population, but did not show a decline in TCT-abnormal ones. It is almost impossible for the human immune system to naturally clear persistent HPV and HR-HPV infections and regresses precancerous lesions within a few months (15). This suggests that the decrease in the HPV infection rate may come from the reduction of new HPV infections. A recent Chinese nationwide research showed that the incidence of STIs was significantly reduced during COVID-19 pandemic (16). The decrease of the five most prevalent STIs was positively linked with COVID-19 pandemic and strict quarantine measures (17).

However, the trend of STIs infection rates during the pandemic is still debatable. Balestri et al. reported that the lockdown measure during the pandemic did not reduce the number of STIs (18). Another study suggested that the incidence of syphilis remained constant, despite strict social distancing and a high risk of SARS-CoV-2 infection (19). This discrepancy was likely due to different data sources of patients with other STIs and the varying degrees of strict isolation policies in different regions during COVID-19 pandemic. Considering the rapid increase of HPV infections after the pandemic, which reached nearly 20%, we strongly recommend that HPV and cervical cancer screening should immediately be reinforced to reduce the dissemination of HPV infection. Furthermore, more practical and feasible HPV and cervical screening strategies should be developed during the pandemic. Recent studies suggested that collecting cervical cancer screening samples at home and mailing them to the hospital for further testing can be an effective method for HPV and cervical cytology screening during the pandemic (20, 21).

The most prevalent genotype during the pandemic was HPV 16, whereas HPV 52 was the dominant genotype before and after the pandemic. Considering that the HPV genotype changed in TCT-negative individuals, but not in TCT-abnormal ones, this suggests that the pandemic has influenced the distribution of HPV genotypes. Potential explanations for this finding include fear of pandemic infection, change of risky sex behavior, different levels of isolation, and outpatient service status, all of which need further investigation. Consistent with a previous study, the most prevalent HPV genotypes found in this study included HPV 52, 58, and 16 before and after the pandemic (22). However, the majority of HPV vaccines only cover HR-HPV 16 and 18, while the 9-valent HPV vaccine is relatively expensive and is not conducive to widespread vaccination in China (23). For effective clinical implementation and application, wider genotype coverage and more affordable HPV vaccines should be developed.

There are several limitations to this study. First, the status of SARS-Cov-2 infection was not confirmed in this study because it was not mandatory for outpatients to be tested for SARS-Cov-2. Second, the impact of confounding factors on HPV infection rates and genotypes distribution such as changes in sexual behavior and reduction in the number of visits should be further investigated to test the reliability of our results (19, 24). Finally, this study only considered the short-term impact of the pandemic on HPV infections in a single center-hospital in Wuhan, China. It is, therefore, necessary to explore the long-term impact of SARS-CoV-2 on HPV infection and other STIs in multicenter studies across different cities and countries.

## Conclusion

Taken together, both the number of cervical screening visits and the HPV infection rate dramatically decreased during COVID-19 pandemic, while the lockdown greatly reduced HPV transmission. After the pandemic has been controlled, the number of cervical cancer screening visits and HPV infection rate rapidly returned to baseline. Therefore, efficient HPV and cervical screening and vaccine programs should be immediately implemented to mitigate HPV transmission and cervical cancer progression.

## Data Availability Statement

The raw data supporting the conclusions of this article will be made available by the authors, without undue reservation.

## Author Contributions

HL and DL designed this study. QY and ZZ analyzed and interpreted the data. HL wrote the manuscript. YL and ZZ revised the manuscript. All authors contributed to the article and approved the submitted version.

## Funding

This study was supported in part by the National Natural Science Foundation of China (Grant No. 81772265).

## Conflict of Interest

The authors declare that the research was conducted in the absence of any commercial or financial relationships that could be construed as a potential conflict of interest.

## Publisher's Note

All claims expressed in this article are solely those of the authors and do not necessarily represent those of their affiliated organizations, or those of the publisher, the editors and the reviewers. Any product that may be evaluated in this article, or claim that may be made by its manufacturer, is not guaranteed or endorsed by the publisher.
