# Supplementary material for: Impact of COVID-19 Outbreak on the Gynecological Outpatients HPV Infection Rate in Wuhan, China: A Retrospective Observational Study
Source: Front Med (Lausanne). 2022 Apr 11;9:799736. doi: 10.3389/fmed.2022.799736 (PMC9035827; doi:10.3389/fmed.2022.799736)
Supplement: Supplementary file 1 [file Data_Sheet_1.pdf]

Table S1 HPV infection rates in overall cohort in different stage population

	Stage I N=4150	Stage II N=4967	Stage III N=2405	Stage IV N=4725	P value
Age(Medium, IQR)	41 (32-49)	41 (32-49)	41 (32-50)	41 (33-50)	0.074
Age 16-35	1443 (34.8)	1730 (34.8)	837 (34.8)	1592 (33.7)	0.158
Age 36-44	1074 (25.5)	1315 (26.5)	567 (23.6)	1217 (25.8)	
Age 45-55	1121 (27.0)	1271 (27.2)	653 (27.2)	1272 (26.9)	
Age >55	529 (12.7)	651 (13.1)	348 (14.5)	644 (13.6)	
Visit per month (Medium, IQR)	739.0 (578.0-812.0)b	812.5 (772.8-885.3)d	371.0 (0.0-641.0)f	798.5 (775.0-819.0)	**<0.001**
HPV	820 (19.8) abc	1155 (23.3) d	369(15.3) f	1003 (21.2)	**<0.001**
HR-HPV	669 (16.1) abc	932 (18.8) d	294 (12.2) f	801 (17.0)	**<0.001**
LR-HPV	255 (6.1)b	370 (7.4) d	103 (4.3) f	331 (7.0)	**<0.001**
M-HPV	185 (4.5) b	277 (5.6) d	66 (2.7) f	246 (5.2)	**<0.001**
S-HPV	635 (15.3) ab	878 (17.7) d	303 (12.6) f	757 (16.0)	**<0.001**
TCT	312 (7.5) a	498 (10.0) de	165 (6.9)	331 (7.0)	**<0.001**
TCT And HPV	180 (4.3) a	295 (5.9) de	84 (3.5)	193 (4.1)	**<0.001**
TCT And HR-HPV	139 (3.3) a	247 (5.0) de	64 (2.7)	166 (3.5)	**<0.001**

Note: HPV: human papillomavirus, HR-HPV: high-risk HPV infection, LR-HPV: Low-risk HPV infection, M-HPV: multiple genotype HPV infection, S-HPV: single genotype HPV infection, TCT: Thin-Prep cytology test abnormal. TCT And HPV: Concurrent of abnormal TCT and HPV infection. a, Stage I Vs. Stage II; b, Stage I Vs. Stage III; c, Stage I Vs. Stage IV; d, Stage II Vs. Stage III; e, Stage II Vs. Stage IV; f, Stage III Vs. Stage IV.

Table S2 HPV infections rates in TCT normal population in different stage.

		Stage I<br>N=3838	Stage II<br>N=4469	Stage III<br>N=2240	Stage IV<br>N=4394	P value
Age(Medium, IQR)		41 (32-49)	41 (32-49)	41 (32-50)	41 (33-49)	0.193
	Age 16-35	1354 (35.3)	1576 (35.3)	795 (35.5)	1513 (34.4)	0.507
	Age 36-44	977 (25.5)	1180 (26.4)	532 (23.8)	1130 (25.7)	
	Age 45-55	1024 (26.7)	1148 (25.7)	605 (27.0)	1174 (26.7)	
	Age >55	483 (12.6)	565 (12.6)	308 (13.8)	577 (13.1)	
HPV		640 (16.4) ab	860 (19.2) d	285 (12.7) f	810 (18.4)	**<0.001**
HR-HPV		530 (13.8)b	685 (15.3)d	230 (10.3)f	635 (14.5)	**<0.001**
LR-HPV		197 (5.1)b	281 (6.3)d	79 (3.5)f	274 (5.6)	**<0.001**
M-HPV		135 (3.5)b	193 (4.3)d	48 (2.1)f	189 (4.3)	**<0.001**
S-HPV		505 (13.2)b	667 (14.9)d	237 (10.6)f	621 (14.1)	**<0.001**

Note: HPV: human papillomavirus, HR-HPV: high-risk HPV infection, LR-HPV: Low-risk HPV infection, M-HPV: multiple genotype HPV infection, S-HPV: single genotype HPV infection, TCT: Thin-Prep cytology test abnormal. a, Stage I Vs. Stage II; b, Stage I Vs. Stage III; c, Stage I Vs. Stage IV; d, Stage II Vs. Stage III; e, Stage II Vs. Stage IV; f, Stage III Vs. Stage IV.

Table S3 HPV infections rates in TCT abnormal population in different stage.

		Stage I N=312	Stage II N=498	Stage III N=165	Stage IV N=331	P value
Age(Medium, IQR)		43 (34-50)	42 (33-51)	46 (35-54)	44 (36-53)	0.196
	Age 16-35	89 (28.5)	154 (30.9)	42 (25.5)	79 (23.9)	0.082
	Age 36-44	80 (25.6)	135 (27.1)	35 (21.2)	87 (26.3)	
	Age 45-55	97 (31.1)	123 (24.7)	48 (29.1)	98 (29.6)	
	Age >55	46 (14.7)	86 (17.3)	40 (24.2)	67 (20.2)	
HPV		180 (57.7)	295 (59.2)	84 (50.9)	193 (58.3)	0.303
HR-HPV		139 (44.6)	247 (49.6)	68 (41.2)	166 (50.2)	0.138
LR-HPV		58 (18.6)	89 (17.9)	24 (14.5)	57 (17.2)	0.724
M-HPV		50 (16.0)	84 (16.9)	18 (10.9)	57 (17.2)	0.281
S-HPV		130 (41.7)	211 (42.4)	66 (40.0)	136 (41.1)	0.954
ASCUS		213 (68.3)	300 (60.2)	96 (58.2)	222 (67.1)	0.054
LSIL		74 (23.7)	139 (27.9)	55 (33.3)	83 (25.1)	
HSIL		25 (8.0)	59 (11.8)	14 (8.5)	26 (7.9)	

Note: HPV: human papillomavirus, HR-HPV: high-risk HPV infection, LR-HPV: Low-risk HPV infection, M-HPV: multiple genotype HPV infection, S-HPV: single genotype HPV infection, TCT: Thin-Prep cytology test abnormal.

Table S4 Prevalence of HPV genotype in HPV abnormal population in different stage.

HPV Genotype	Overall Cohort					TCT positive				
	Stage I N=4151	Stage II N=4967	Stage III N=2405	Stage IV N=4725	P value	Stage I N=312	Stage II N=498	Stage III N=165	Stage IV N=331	P value
HR-HPV										
HPV16	116 (2.79)	116 (2.34)	58 (2.41)	120 (2.54)	0.555	33 (10.9)a	37 (7.4)	18 (10.9)	34 (10.3)	0.317
HPV18	41 (0.99)	58 (1.17)	14 (0.58)	38 (0.80)	0.067	14 (4.5)	15 (3.0)	3 (1.8)	9 (2.7)	0.387
HPV26	1 (0.02)	0 (0.00)	0 (0.00)	1 (0.02)	0.642	0 (0.0)	0 (0.0)	0 (0.0)	0 (0.0)	NA
HPV31	11 (0.27)	26 (0.52)	7 (0.29)	21 (0.44)	0.191	2 (0.6)	5 (1.0)	3 (1.8)	3 (0.9)	0.669
HPV33	23 (0.55)	32 (0.64)	13 (0.54)	30 (0.63)	0.909	4 (1.3)	9 (1.8)	3 (1.8)	5 (1.5)	0.940
HPV35	12 (0.29)	16 (0.32)	4 (0.17)	14 (0.30)	0.691	5 (1.6)	7 (1.4)	1 (0.6)	3 (0.9)	0.729
HPV39	25 (0.60)a	72 (1.45)	19 (0.80)	43 (0.91)	**<0.001**	2 (0.6)	12 (2.4)	7 (4.2)	6 (1.8)	0.061
HPV45	6 (0.14)	10 (0.20)	3 (0.12)	6 (0.13)	0.775	0 (0.0)	2 (0.4)	1 (0.6)	1 (0.3)	0.661
HPV51	39 (0.94)	58 (1.67)	13 (0.54)	47 (0.99)	0.140	9 (2.9)	22 (4.4)	4 (2.4)	8 (2.4)	0.343
HPV52	139 (3.35)a	236 (4.75)d	56 (2.33)f	195 (4.13)	**<0.001**	33 (10.6)	56 (11.2)	8 (4.8)	39 (11.8)	0.089
HPV53	90 (2.17)	124 (2.50)	45 (1.87)	116 (2.46)	0.304	19 (6.1)	33 (6.6)	11 (6.7)	23 (6.9)	0.978
HPV56	50 (1.20)	62 (1.25)	25 (1.04)	65 (1.38)	0.673	12 (3.8)	23 (4.6)	8 (4.8)	20 (6.0)	0.622
HPV58	118 (2.84)	153 (3.08)d	46 (1.91)	125 (2.65)	**0.034**	30 (9.6)	50 (10.0)	12 (7.3)	30 (9.1)	0.758
HPV59	43 (1.04)	52 (1.05)	23 (0.96)	50 (1.06)	0.981	10 (3.2)	15 (3.0)	5 (3.0)	11 (3.3)	0.995
HPV66	59 (1.42)bc	44 (0.86)	15 (0.62)	37 (0.78)	**0.004**	12 (3.8)	13 (2.6)	5 (3.0)	9 (2.7)	0.772
HPV68	21 (0.51)	34 (0.68)	12 (0.50)	25 (0.53)	0.613	7 (2.2)	10 (2.0)	2 (1.2)	3 (0.9)	0.506
HPV82	9 (0.22)	23 (0.46)	3 (0.12)	15 (0.32)	0.054	0 (0.0)	4 (0.8)	0 (0.0)	1 (0.3)	0.241
LR-HPV										
HPV6	30 (0.72)	40 (0.81)	12 (0.50)	43 (0.91)	0.292	6 (1.9)	14 (2.8)	7 (4.2)	8 (2.4)	0.503
HPV11	21 (0.51)	21 (0.42)	3 (0.12)	14 (0.30)	0.066	3 (1.0)	8 (1.6)	1 (0.6)	3 (0.9)	0.655
HPV40	5 (0.12)	3 (0.06)	0 (0.00)	1 (0.02)	0.137	0 (0.0)	0 (0.0)	0 (0.0)	0 (0.0)	NA
HPV42	15 (0.36)	23 (0.46)	3 (0.12)	16 (0.34)	0.149	2 (0.6)	9 (1.8)	2 (1.2)	6 (1.8)	0.525
HPV43	43 (1.00)	39 (0.79)	18 (0.79)	47 (0.99)	0.445	8 (2.6)	12(2.4)	3 (1.8)	7 (2.1)	0.951
HPV44	15 (0.36)ac	50 (1.00)d	8 (0.33)f	50 (1.06)	**<0.001**	7 (2.2)	8 (1.6)	0 (0.0)	6 (1.8)	0.310
HPV55	21 (0.51)	36 (0.72)	12 (0.50)	37 (0.78)	0.273	6 (1.9)	5 (1.0)	4 (2.4)	6 (1.8)	0.574
HPV61	57 (1.37)	96 (1.93)	33 (1.37)	88 (1.86)	0.086	13 (4.2)	20 (4.0)	7 (4.2)	21 (6.3)	0.422
HPV81	61 (1.47)	89 (1.79)	19 (0.79)f	76 (1.61)	**0.010**	13 (4.2)	20 (4.0)	2 (1.2)	12 (3.6)	0.355
HPV83	5 (0.12)	7 (0.14)	2 (0.08)	10 (0.21)	0.529	2 (0.6)	3 (0.6)	0 (0.0)	3 (0.9)	0.684

Note: HPV: human papillomavirus, HR-HPV: high-risk HPV infection, LR-HPV: Low-risk HPV infection. a, Stage I Vs. Stage II; b, Stage I Vs. Stage III; c, Stage I Vs. Stage IV; d, Stage II Vs. Stage III; e, Stage II Vs. Stage IV; f, Stage III Vs. Stage IV.
